# Lipid Peroxidation and Total Cholesterol in HAART-Naïve Patients Infected with Circulating Recombinant Forms of Human Immunodeficiency Virus Type-1 in Cameroon

**DOI:** 10.1371/journal.pone.0065126

**Published:** 2013-06-07

**Authors:** Georges Teto, Georgette D. Kanmogne, Judith N. Torimiro, George Alemnji, Flore N. Nguemaim, Désiré Takou, Aubin Nanfack, Asonganyi Tazoacha

**Affiliations:** 1 Laboratory of Immunology, Biochemistry and Biotechnology, University of Yaoundé I, Yaoundé, Cameroon; 2 CIRCB (Chantal Biya International Research Center), Yaoundé, Cameroon; 3 Faculty of Medicine and Biomedical Sciences, University of Yaoundé I, Yaoundé, Cameroon; 4 Department of Pharmacology and Experimental Neuroscience, University of Nebraska Medical Center, Omaha, Nebraska, United States of America; IPO, Inst Port Oncology, Portugal

## Abstract

**Background:**

HIV infection has commonly been found to affect lipid profile and antioxidant defense.

**Objectives:**

To determine the effects of Human Immunodeficiency Virus (HIV) infection and viral subtype on patient’s cholesterol and oxidative stress markers, and determine whether in the absence of Highly Active Antiretroviral Therapy (HAART), these biochemical parameters could be useful in patient’s management and monitoring disease progression in Cameroon. For this purpose, we measured total cholesterol (TC), LDL cholesterol (LDLC), HDL cholesterol (HDLC), total antioxidant ability (TAA), lipid peroxidation indices (LPI), and malondialdehyde (MDA) in HIV negative persons and HIV positive HAART-naïve patients infected with HIV-1 group M subtypes.

**Methods:**

We measured serum TC, LDLC, HDLC, plasma MDA, and TAA concentrations, and calculated LPI indices in 151 HIV-positive HAART-naïve patients and 134 seronegative controls. We also performed gene sequence analysis on samples from 30 patients to determine the effect of viral genotypes on these biochemical parameters. We also determined the correlation between CD4 cell count and the above biochemical parameters.

**Results:**

We obtained the following controls/patients values for TC (1.96±0.54/1. 12±0. 48 g/l), LDLC (0. 67±0. 46/0. 43±0. 36 g/l), HDLC (105. 51±28. 10/46. 54±23. 36 mg/dl) TAA (0. 63±0. 17/0. 16±0. 16 mM), MDA (0. 20±0. 07/0. 41±0. 10 µM) and LPI (0. 34±0. 14/26. 02±74. 40). In each case, the difference between the controls and patients was statistically significant (p<0.05). There was a positive and statistically significant Pearson correlation between CD4 cell count and HDLC (r = +0.272; p<0.01), TAA (r = +0.199; p<0.05) and a negative and statistically significant Pearson correlation between CD4 cell count and LPI (r = −0.166; p<0.05). Pearson correlation between CD4 cell count and TC, CD4cell count and LDLC was positive but not statistically significant while it was negative but not statistically significant with MDA. The different subtypes obtained after sequencing were CRF02_AG (43.3%), CRF01_AE (20%), A1 (23.3%), H (6.7%), and G (6.7%). None of the HIV-1 subtypes significantly influenced the levels of the biochemical parameters, but by grouping them as pure subtypes and circulating recombinant forms (CRFs), the CRF significantly influenced TC levels. TC was significantly lower in patients infected with CRF (0.87±0.27 g/l) compared to patients infected with pure HIV-1 subtypes (1.32±0.68 g/l) (p<0.017). MDA levels were also significantly higher in patients infected with HIV-1CRF01_AE (0.50±0.10 µM), compared to patients infected with CRF02_AG (0. 38±0. 08 µM) (p<0.018).

**Conclusion:**

These results show that HIV infection in Cameroon is associated with significant decrease in TAA, LDLC, HDLC and TC, and increased MDA concentration and LPI indices which seem to be linked to the severity of HIV infection as assessed by CD4 cell count. The data suggests increased oxidative stress and lipid peroxidation in HIV-infected patients in Cameroon, and an influence of CRFs on TC and MDA levels.

## Introduction

Human immunodeficiency virus type 1 (HIV-1) is the pathogen responsible for acquired immunodeficiency syndrome, a disease which has spread throughout the world and which affects immune cells, especially CD4+ lymphocytes and macrophages [1]. About 68% of HIV-infected individuals live in sub Saharan Africa [2], one of the most impoverished regions of the world; this represents two third of 34 millions individuals currently living with HIV/AIDS [3], [2]. In Cameroon the prevalence of HIV infection is estimated at 5. 5% [4], while antiretroviral therapy (ART) coverage is below 40% [2], suggesting that about 60% of HIV-infected Cameroonians in need of treatment do not have access to ART. For these patients, monitoring of biochemical parameters such as nutritional status and oxidative stress markers could help in the management of HIV/AIDS patients.

HIV-1 is divided into four groups: M for major, O for outlier, N for non M non O [6], [7], and P [5]. HIV-1 group M viruses are further divided into nine pure subtypes and about 54 circulating recombinant forms (CRF) [8], [9]; CRF02_AG subtypes are predominant in West and Central Africa while CFR01_AE subtypes are present in Central Africa, Thailand and other Asian countries [10], [11]. All these groups and subtypes are present in countries where HIV-1 has been implicated in many biochemical disorders among which dyslipidemia and antioxidant imbalance [12], [13].

Dyslipidemia is a clinical condition which often leads to alterations in lipid profile: total cholesterol (TC), low density lipoprotein cholesterol (LDLC) and high density lipoprotein cholesterol (HDLC) [14], [15]. Antioxidant imbalance which is assessed through plasma malondialdehyde concentration and plasma total antioxidant ability, is a condition which can contribute to increased destruction of CD4+ T cells and disease progression if the balance is in favor of pro-oxidant (free radicals) generation [16], [17]. Deficiency in antioxidants in many HIV/AIDS patients may also potentiate the harmful effects of free radical action and accelerate disease progression [18], [12].

Cameroon is a country with a high diversity of HIV-1 subtypes [19], [20] but the clinical implications of this multitude of HIV-1 subtypes has not previously been investigated. The objective of this study was to determine the effects of HIV-1 infection and viral subtype on patient’s cholesterol (TC, LDLC, HDLC), lipid peroxidation indices (LPI), and oxidative stress markers [total antioxidant ability (TAA) and malondialdehyde (MDA)], to determine whether in the absence of HAART, these biochemical parameters could be useful in patient’s management and monitoring disease progression.

## Subjects and Methods

### Subjects

Informed consent was obtained from all subjects according to the guidelines of the Cameroon National Ethics Committee that approved the study. After obtaining informed consent, we enrolled 285 individuals who met our inclusion criteria: (1) for control subjects, exclusion criteria were pregnancy, serological evidence of hepatitis B/C, diabetes, hypertension, current intake of drugs, alcohol, tobacco, malaria and other known parasitic infection and inclusion criteria were HIV negative with none of the above conditions, and be able to read and sign an informed consent; (2) for patients, the exclusion criteria were the same as for control subjects; in addition, HIV-positivity was confirmed.

The 285 individuals included 151 patients (thirty were taken for genotypic studies) and 134 control subjects.

### Sample Collection

Following a 10 hour fast, 10 ml of blood was collected from each participant into a labeled dry tube (5 ml) and a tube containing EDTA anticoagulant (5 ml). Following clotting, the dry tubes were centrifuged at 1200 g for 15 min to collect serum which was aliquoted and used for the assay of the different biochemical parameters while EDTA tubes were centrifuged at the same conditions to collect plasma which was also aliquoted and used for RNA extraction and measurement of oxidative stress markers. All samples were stored at −20°C and processed within four days after collection.

#### 1) Quantification of biochemical parameters

Serum TC, HDLC, LDLC concentrations were determined using commercially available kits (Human Gesellschaft fur Biochemica und DiagnosticambH Kit, Max-Planck-Ring 21-Wiesbaden-Germany). Plasma TAA was determined using the method of Benzie and Strain [21], while MDA was determined using the method of Kohn and Liversedge [22] as described by Lefèvre [23]. MDA measurement is based on a reaction between malondialdehyde and thiobarbituric acid which forms a pink pigment that absorbs at 532 nm at 90°C −100°C at pH 2, while TAA is measured using the ferric reducing ability of plasma (FRAP) based on the ability of the antioxidants present in plasma to reduce Fe^3+^ to Fe^2+^ at pH 3.6 in the presence of 2.4.6 tri (−2 pyridyl-s-triazine); the reaction produces an intense blue color that absorbs at 593 nm. LPI was obtained by calculating the ratio MDA/TAA.

TC concentration was determined using colorimetric enzymatic techniques based on the successive action of cholesterol oxidase and peroxidase; HDLC concentration in the serum supernatant was determined by the same process after the precipitation of VLDL cholesterol, LDL cholesterol and chylomicrons in the presence of phosphotungstic acid and MgCl_2_. Results were calculated using the formula:

TC (g/l) or HDLC (mg/dl) concentrations = (OD_500nm_ sample/OD_500nm_ standard) × Concentration of standard (essentially as recommended by the manufacturer in the kits).

LDLC concentration was determined using the formula of Friedewaldet *al*. [24]:

LDLC (mg/dl) = TC (mg/dl)-[HDLC (mg/dl)-Triglycerides (mg/dl)/5].

#### 2) RNA extraction and PCR amplification

RNA was extracted from patient’s plasma using the QIAmp Viral RNA kit ([Qiagen, Hilden, Germany]), according to the manufacturer’s instructions.

RNA and cDNA samples were then amplified in a one tube, two steps RT- PCR using a thermal cycler (TECHNE TC 412[TECHNE Inc, Burlington, New Jersey, USA]) with the following primers: H1G777 (TCACCTAGAACTTTGAATGCATGGG) sense (nucleotide 777 to 801 of HIV-1 genome) and H1P202 (CTAATACTGTATCATCTGCTGCTCCTGT) antisense (nucleotide 1874 to 1898 of HIV-1 genome). Nested PCR was performed using H1Gag1584 (AAAGATGGATAATCCTGGG) sense and g17 (TCCACATTTCCAACAGCCCTT) antisense primers to enable amplification of the 460 bp encoding amino acid 132 of p24 to amino acid 40 of p7 from the gag gene. The amplification conditions of the RT-PCR were as followed: 50 min at 42°C (cDNA reaction) followed by 3 min at 94°C and the addition of 0, 2 µl Taq polymerase (5UI/ µl); then the reaction continued with 40 cycles of 30s, 30s, and 90s at 94°C, 50°C, and 72°C, respectively, and 1 cycle of 7 min at 72°C. For Nested PCR, cycling conditions were 1 cycle of 2 min at 94°C; 35 cycles of 30s, 30s, and 60s at 94°C, 50°C, and 72°C respectively; and 1cycle of 7 min at 72°C. The PCR amplification products were detected by electrophoresis on a 1% agarose gel and visualized by ethidium bromide staining under UV light.

#### 3) DNA sequencing

**Figure 1:**
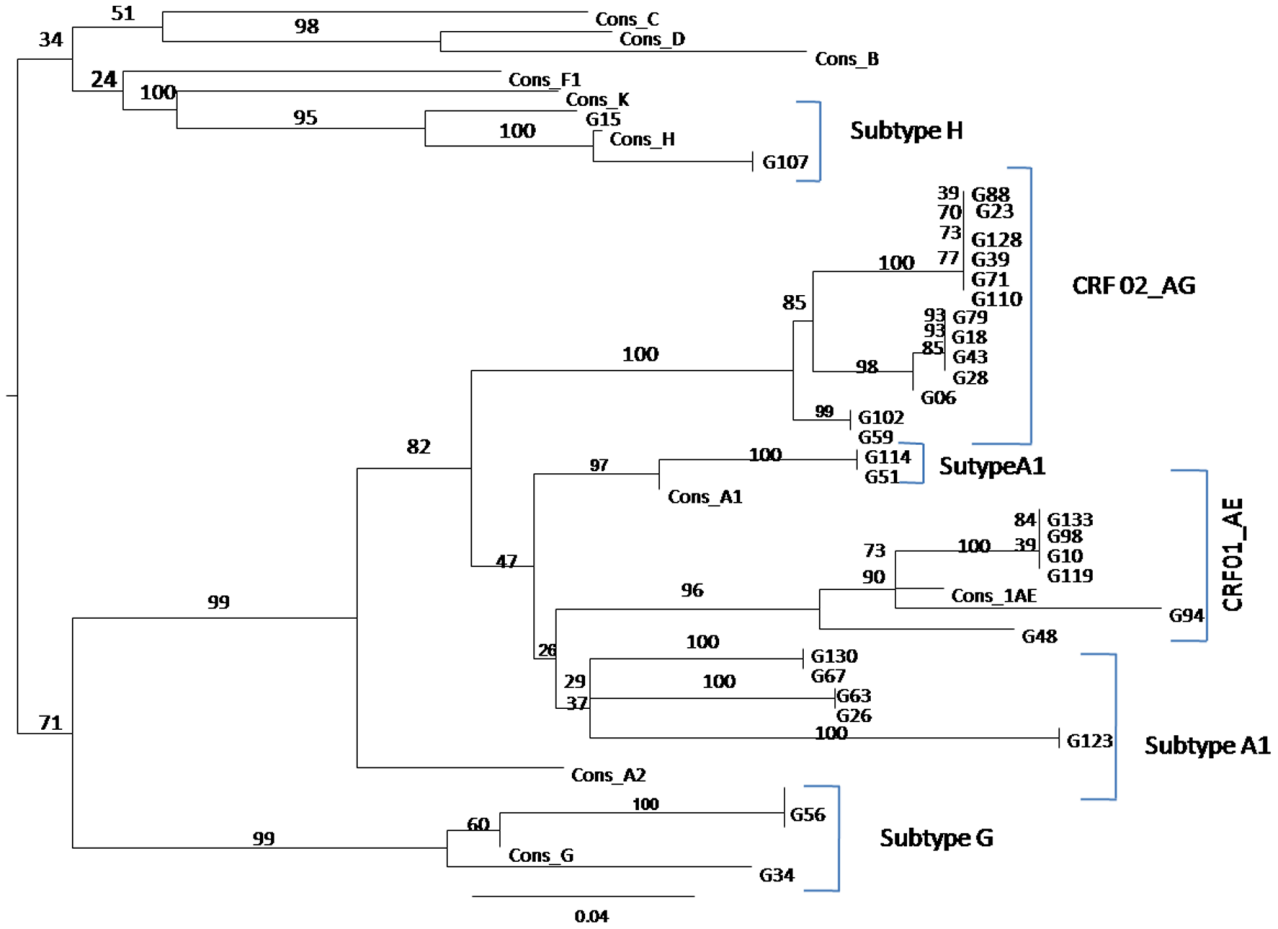
Phylogenetic tree of the different subtypes of HIV-1 group M included in the study (*460 bp encoding amino acid 132 of p24 to amino acid 40 of p7 from the gag gene)*. Cons = reference sequences; G = sample.

The 460 bp fragments obtained were sequenced using the previously described primers H1Gag 1584 and g17 with the same PCR amplification program [11]. Nucleotide sequences were obtained by direct sequencing of the PCR products. The amplified DNA was purified using an AmiconMicrocon Ultra pure kit (centrifugal filters devices-Millipore) and directly sequenced using Big-Dye chemistry (Perkin-Elmer). Electrophoresis and data collection were done on an Applied Biosystems 3130 XL automatic DNA sequencer. Nucleotide sequences were aligned using CLUSTAL W [25], with minor manual adjustments as appropriate for the DNA sequences. Regions that could not be aligned unambiguously, due to sequence variability or length, were omitted from the analysis. The phylogenetic tree (Figure 1) was generated by the neighbor-joining method [26] and reliability of the branching orders determined by the bootstrap approach [27]. The CLUSTAL W. Genetic distances were calculated using the Kimura’s two-parameter method [28].

### Statistical Analysis

Data were analyzed using PASW STATISTICS version 18 software. We obtained means, standard deviation and percentages. Two-group comparisons were done with the parametric Student t test or the non parametric Mann Whitney test, and ANOVA was used when more than two series of data were compared. Kruskal Wallis test was used for quantitative variables while *X^2^* test was used for qualitative variables. Pearson (parametric) or Spearman (non parametric) correlations were used to establish the correlation between the different parameters. Logistic regression and ANOVA were used to study the association of the different subtypes with biochemical parameters.

Results were considered statistically significant at p<0.05.

## Results

### Participants’ Demographics and Clinical Characteristics

Participant’s demographics characteristics are summarized in Table 1. A total of 285 subjects (151 HIV+ and 134 seronegative controls) were evaluated in this study. Of the HIV+ group, 55 (36.4%) were male and 96 (63.6%) were female. Of the 134 subjects in the control group, 73 (54.5%) were male and 61 (45.5%) were female. The average ages were 35.5±9.32 years for HIV+ group and 27.5±7.70 years for the control group. Of the 151 HIV+ cases, 15 (10%) were asymptomatic, while 136 (90%) had experienced at least one AIDS event based on the occurrence of opportunistic infections (prurigo in 43 cases, cryptococcosis in 8 cases, Kaposi sarcoma in 8 cases, cytomegalovirus infection in 10 cases, toxoplasmosis in 10 cases, pneumocystosis in 24 cases, and tuberculosis in 33 cases). Based on the Centers for Disease Control (CDC) AIDS classification criteria [29], the patients belonged to category A (10%), category B (51.65%) and category C (38. 41%). Increase in LPI and MDA and decrease in TC, HDLC, LDLC, TAA are linked to reduction in CD4 cell counts in a statistically significant manner (Table 2). There was a positive and statistically significant Pearson correlation between CD4 cell count and HDLC (r = +0.272; p<0.01) and TAA (r = +0.199; p<0.05) and a negative and statistically significant Pearson correlation between CD4 cell count and LPI (r = −0.166; p<0.05). Pearson correlation between CD4 cell count and TC and LDLC was positive but not statistically significant while it was negative and not statistically significant with MDA (Table 3).

**Table 1:** Demographics and clinical characteristics of participants.

Characteristics	HIV+ Patients	HIV-Controls	P
Total number	(N = 151)	(N = 134)	
Sex (% female)	63.6	45.5	0.0001
Age (mean ± SD)	35.58±9.32	27.65±7.70	0.0001
Age range	16–56	16–56	
Education (mean years ± SD)	12.20±1.68	12.50±1.57	0.71
AIDS (%)	38.41		

**Table 2:** Biochemical parameters in HIV-infected patients, stratified according to CD4 cell count, compared with control subjects.

Parameters	HIV-Controls	HIV+	Patients	(Cell/µL)	P
		≥500	200–499	<200	
		(A_1_)	(B_2_)	(C_3_)	
	N = 134	N = 15	N = 78	N = 58	
TC (g/l)	1,96±0,54	1,18±0,55	1,07±0,38	0,97±0,36	0.0001
LDLC (g/l)	0, 67±0, 46	0,29±0,21	0,50±0,42	0,37±0,26	0.0001
HDLC (mg/dl)	105, 51±28, 10	46,91±25,22	46,51±21,56	45,27±26,45	0.0001
TAA (mM)	0, 63±0, 17	0,27±0,26	0,17±0,14	0,13±0,13	0.0001
MDA (µM)	0, 20±0, 07	0,39±0,10	0,41±0,11	0,42±0,10	0.0001
LPI	0, 34±0, 14	17,53±32,83	30,83±96,87	31,41±90,51	0.0001

*Every value is the mean ± standard deviation.* P value: statistically significant difference between each clinical category and HIV-controls group for each biochemical marker mean value. (A_1_), (B_2_), (C_3_): Clinical categories.

**Table 3:** Biochemical parameters in HIV-infected patients, correlated with CD4 using Pearson correlation coefficient.

	CD_4_	TC	HDLC	LDLC	TAA	MDA	LPI
CD_4_	1						
TC	0,037	1					
HDLC	0,274**	0,583**	1				
LDLC	0, 065	0,530**	0,142	1			
TAA	0,199*	0,042	0,032	0,018	1		
MDA	−0,059	−0,035	−0,035	−0,022	0,074	1	
LPI	−0,166*	−0,079	−0,066	−0,030	−0,968**	0,125	1

* Significant Pearson correlation (P<0, 05 at a bilateral level).

** Significant Pearson correlation (p<0, 01 at a bilateral level).

### HIV Genotyping

Samples from 50 HIV+ patients were used in genotypic studies, and we successfully sequenced the viral genome in samples from 30 patients, all of which belonged to the CDC category B [29]. Results indicated that 43.3% were HIV-1 CRF02_AG, 20% CRF01_AE; 23.3% subtype A1, 6.7% subtype H, and 6.7% subtype G (Table 4).

**Table 4:** Distribution of HIV-1 subtypes in patients by sex and CD_4_ cell counts.

		Men			Women			Total (%)
		CD4 cells count/µl			CD4 cellscount/µl			
		≥500	200–499	<200	≥500	200–499	<200	
SUBTYPES	CRF01_AE	0	2	0	0	4	0	6 (20.0%)
	CRF02_AG	1	3	2		5	2	13(43.3%)
	A1	0	4	2	0	0	1	7 (23.3%)
	G	0	0	1	0	1	0	2 (6.7%)
	H	0	1	0	0	1	0	2 (6.7%)
	CRFs	1	5	2	0	9	2	19(63.3%)
	Pure	0	5	3	0	2	1	11(36.6%)
Total number of subjects		1	10	5	0	11	3	

### Biochemical Parameters and HIV-1 Subtypes Effects

Results in Table 4 show that CRF02 _AG subtype is the most frequent (43, 3%) followed by A1 (23, 3%), CRF01 _AE (20%), G (6, 7%) and H (6, 7%) subtypes. CRF02 _AG and CRF01 _AE subtypes were the most frequent in women compared to men; every HIV-1 subtype represented here is implicated in at least one class of CD4 cells count in men as well as in women.

Results for TC, LDLC, HDLC, TAA, MDA, and LPI are summarized in Table 5. There was a statistically significant difference (p<0.05) between patients and controls for TC, LDLC, HDLC, TAA, MDA, and LPI. MDA (an oxidative stress marker), and LPI mean values are higher in patients compared to controls while TC, LDLC, HDLC, TAA mean values are lower in patients compared to controls (Table 5); there was a statistically significant Pearson positive correlation (p<0.01 at a bilateral level) between TC and LDLC (r = 0.530); TC and HDLC (r = 0.583) and a statistically significant Pearson negative correlation (p<0.01 at a bilateral level) between TAA and LPI (r = −0.968). The Pearson correlation between TC and MDA was negative and non significant (r = −0.035).

**Table 5:** Comparison of different biochemical parameters between patients and controls.

Parameters	Controls±SD	Patients±SD	P
TC (g/l)	1.96±0.54	1. 12±0. 48	0.0001
LDLC (g/l)	0. 67±0. 46	0. 43±0. 36	0.0002
HDLC (mg/dl)	105. 51±28. 10	46. 54±23. 36	0.0001
TAA (mM)	0. 63±0. 17	0. 16±0. 16	0.0001
MDA (µM)	0. 20±0. 07	0. 41±0. 10	0.0002
LPI	0. 34±0. 14	26. 02±74. 40	0.0001

Every value is the mean ± standard deviation. SD = Standard deviation.

Results for the effect of HIV subtype on TC are summarized in Table 6. There was a statistically significant difference in the level of TC in patients infected with CRFs (CRF02 _AG and CRF01 _AE) and pure HIV-1 subtypes (G, H and A_1_) (p = 0.017); there was a lower mean value in CRFs patient group (0.87±0. 27 g/l) compared to patients carrying pure subtypes group (1. 32±0. 68 g/l). Patients carrying CRFs had lower LDLC, HDLC, TAA mean values compared to patients carrying the pure subtypes although the results were not statistically significant (Table 6). Before grouping the different subtypes, we first looked at the implication of each subtype taken alone in men as well as in women on each biochemical parameter using both a logistic regression test and ANOVA, but results showed no statistically significant difference between groups (data not shown).

**Table 6:** Comparison of different biochemical parameters between patients infected with CRF CRF01_AE, CRF02_AG and pure HIV1 subtypes (G, H, and A1).

Parameters	Subtypes	Mean ± SD	P	Subtypes	Mean ± SD	P
TC (g/l)	(CRF)	0.87±0.27	0.017	CRF01 _AE	1.74±0.97	0.69
	(G, H and A_1_)	1.32±0.68		CRF02 _AG	1.13±0.41	
HDLC (mg/dl)	(CRF)	41.18±22.76	0.68	CRF01 _AE	54.17±22.57	0.22
	(G, H and A_1_)	44.74±22.57		CRF02 _AG	40.38±22.07	
LDLC (g/l)	(CRF)	0.33±0.18	0.059	CRF01 _AE	0.88±0.81	0.11
	(G, H and A_1_)	0.60±0.53		CRF02 _AG	0.47±0.27	
TAA (mM)	(CRF)	0.09±0.07	0.169	CRF01 _AE	0.10±0.11	0.61
	(G, H and A_1_)	0.13±0.12		CRF02 _AG	0.14±0.12	
MDA (µM)	(CRF)	0.44±0.12	0.51	CRF01 _AE	0.50±0.10	0.018
	(G, H and A_1_)	0.41±0.10		CRF02 _AG	0.38±0.08	
LPI	(CRF)	23.92±52.31	0.92	CRF01 _AE	59.22±123.09	0.16
	(G, H and A_1_)	25.99±69.74		CRF02 _AG	10.65±13.29	

Every value is the mean ± standard deviation.

Further, the results for the effect of HIV subtypes on MDA, TC, LDLC, HDLC and LPI are shown in Table 6. There was a statistically significant difference in MDA levels in patients with the CRF01 _AE subtype (1.32±0.68 µM) compared to patients infected with CRF01 _AG subtype (0.38±0. 08 µM) (p = 0.018). Levels of TC, LDLC, HDLC and LPI in patients infected with the CRF01 _AE subtype were higher compared to patients infected with the CRF01 _AG subtype, although the differences were not statistically significant. In general, the CRF01 _AE subtype seemed to induce higher lipid peroxidation. We performed additional analyses to determine whether HIV-1 subtypes A1, G, and H influenced the levels of the different biochemical parameters, but results showed no statistically significant difference (data not shown).

## Discussion

Transport of cholesterol in the organism is by low density lipoproteins (LDL; 70%), high density lipoproteins (HDL, 20 to 35%) and by very low density lipoproteins (VLDL, 5 to 12%) [30]. LDL-cholesterol is implicated in the genesis of atherosclerosis, while HDL-cholesterol facilitates the elimination of excess lipids from cells to liver; high LDL-cholesterol or low HDL cholesterol is associated with coronary heart disease [31].

This study showed statistically significant (p<0.05) lower mean values of TC, HDLC and LDLC in HIV-infected patients compared to serologically negative controls even though their mean ages and sex distribution were somewhat different (Table 1). Age is a major factor in the amount of cholesterol in blood for men older than 45 years and women older than 55 years [32]. Raisonnier and *al.* [33] showed in healthy Cameroonians that HDLC concentration varied with gender but not with age. Our present study showed no significant effect of age on the biochemical markers analyzed (data not shown). Thus, it is likely that the increased oxidative stress and lipid peroxidation observed in our study is directly due to HIV infection or viral-induced lipodystrophy, as lipodystrophy in HIV infection is associated with dyslipidemia [34], [35]. Our data are in agreement with previous studies that showed lower TC, LDLC and HDLC in HIV-1 infected patients [36], and demonstrated that HIV-induced dyslipidemia was associated with lower HDLC and LDLC [37].

TAA was evaluated using the FRAP test which expresses the antioxidant potential of the organism. It measures its capacity to neutralize through antioxidant molecules the oxidant (free radicals) damage on various substrates (proteins, lipids, carbohydrates, nucleic acids). Our results showed about a threefold reduction of TAA plasma concentration in patients compared to controls. This may be linked to the high level of free radicals production due to the antigenic (virus) activation of lymphocytes, phagocytes and chronic inflammatory processes induced by viral replication [38]. The reactive oxygen species (^−^OH, HO**^.^**, O^−^_2_, H_2_O_2_) produced during chronic inflammation react with antioxidants and contribute to the reduction of their plasma concentration [13]. This results in the antioxidant/pro-oxidant balance altered in favor of pro-oxidant; which leads to severe lipids peroxidation and cells apoptosis as reflected by the high concentration of MDA in HIV-infected patients (our results and refs [39], [13]).

Chronic inflammation in HIV infection increase free radicals formation; these free radicals induce lipid peroxidation which leads to MDA formation [40]. Our results also showed high plasma MDA concentration in patients compared to controls (Table 7); these results are in agreement with those reported by Djinhi and collaborators [41]. The high plasma MDA concentrations we report here are probably the consequence of the effects of free radicals on polyunsaturated lipids which induce oxidative stress, and produces destructive effects such as cells apoptosis, a major cause of CD4 cell depletion during HIV infection, particularly in the early stage of the infection [39], [42]. We established a negative Pearson correlation between MDA and TC (r = −0.035), meaning that MDA plasma concentration increases with the decrease of total cholesterol due to lipids peroxidation.

**Table 7:** Comparison of plasma MDA, TC, HDLC, LDLC concentrations by sex in controls and patients group.

Parameters	Groups	Men	women	P
MDA (µM)	Patients	0,43±0,10	0,39±0,10	0.68
	Controls	0,26±0,04	0,14±0,03	0.019
HDLC (mg/dl)	Patients	42,12±22,66	49,07±23,5	0.001
	Controls	101,99±28,69	109,72±27,01	0.001
LDLC (g/l)	Patients	0,38±0,25	0,46±0,40	0.60
	Controls	0,63±0,42	0,72±0,51	0.061
TC (g/l)	Patients	1,02±0,41	1,17±0,51	0.021
	Controls	1,89±0,48	2,05±0,59	0.024

Every value, except P values, is the mean ± standard deviation.

Lipid peroxidation indice (LPI) is the ratio MDA/TAA; it estimates the degree of free radical aggression due to HIV infection. When the plasma total antioxidant ability decreases or when the plasma MDA concentration increases, LPI increases and this is associated with increased oxidative stress in patients [38]. Our current study show a 76-fold increase in LPI in patients compared to the controls, which is in agreement with previous studies [38] and show that in HIV infection in Cameroon is associated with increased production of free radicals, considerable decrease in TAA, and increased oxidative stress. It has also been established that HIV-1 uses available antioxidants for its replication and this phenomenon adds to the chronic inflammatory process that speeds up CD4 cells apoptosis and disease progression [36]. Generation of free radicals and certain cytokines (TNFα, IL_1_) are thought to be implicated in the decrease of TAA, LDLC, HDLC and TC and the increase of MDA and LPI [43], [41], [14].

Our patients were infected by different HIV-1 subtypes, as determined by the sequencing experiments (Table 4). All HIV-1 subtypes, circulating recombinant forms (CRFs), as well as pure subtypes are implicated in disease progression, although some subtypes like D were established to be more implicated than others due to their dual tropism [44].

Our results (Table 6), in spite of the small sample size, seem to indicate that CRFs may have aggravating effects on lipodystrophy since they leads to dyslipidemia in HIV infected patients [45]. We also show high plasma MDA concentration and higher levels of LDLC in the CRFs group than in the pure subtype group. This could be an indication of an elevated free radicals generation in CRFs infected patients, thus explaining the low serum TC, HDLC and LDLC concentrations due to lipid peroxidation. These results are in conformity with those of other authors [45], [39], [13], [15].

The CRF01 _AE subtype seems to induce high lipid peroxidation (Table 6), probably due to its replication velocity, since high concentrations of free radicals are produced during HIV-1 replication process [46]. Free radicals formation may also enhance HIV replication in T cells and macrophages by acting on the transcription factor NF-κB, [46], [17]; and HIV replication stimulates cytokine production, particularly the tumor necrosis factor alpha (TNFα) which in turn stimulates free radical generation [41]. It has also been established that during HIV replication, HIV infected cells express different proteins (kinases, transport proteins, receptors, chaperons molecules), some of which were identified to be responsible for free fatty acids synthesis, lipids oxidation, alteration in lipid metabolism, and lipid transport deregulation [37]. Our future studies will determine whether any of these viral-induced kinases, receptors or chaperons is responsible for the high lipid peroxidation and increased oxidative stress in our HIV-infected population.

### Conclusion

These results, in spite of some limitations like mean age and sex distribution differences, and small sample size for genotyping studies, show a significant reduction in TAA, LDLC, HDLC, TC and an elevated MDA concentration and LPI in HIV-positive patients compared to serologically negative controls. This may be due to chronic inflammation caused by HIV replication which produces free radicals. These free radicals may be responsible for the lipids peroxidation, CD4 cell reduction, low TAA, and high LPI and MDA observed in our study. The differences in biochemical parameters in patients infected with different HIV subtypes may be due to their replication velocities as HIV-1 CRF01 _AE has been shown to have a faster replication velocity [46].
